# Connectome-Based Model Predicts Deep Brain Stimulation Outcome in Parkinson's Disease

**DOI:** 10.3389/fncom.2020.571527

**Published:** 2020-10-28

**Authors:** Ruihong Shang, Le He, Xiaodong Ma, Yu Ma, Xuesong Li

**Affiliations:** ^1^School of Computer Science and Technology, Beijing Institute of Technology, Beijing, China; ^2^Department of Biomedical Engineering, Center for Biomedical Imaging Research, School of Medicine, Tsinghua University, Beijing, China; ^3^Center for Magnetic Resonance Research, University of Minnesota, Minneapolis, MN, United States; ^4^Department of Neurosurgery, Tsinghua University Yuquan Hospital, Beijing, China

**Keywords:** deep brain stimulation (DBS) surgery, Parkinson's disease, machine learning, brain network, rs-fMRI

## Abstract

Subthalamic nucleus deep brain stimulation (STN-DBS) is an effective invasive treatment for advanced Parkinson's disease (PD) at present. Due to the invasiveness and cost of operations, a reliable tool is required to predict the outcome of therapy in the clinical decision-making process. This work aims to investigate whether the topological network of functional connectivity states can predict the outcome of DBS without medication. Fifty patients were recruited to extract the features of the brain related to the improvement rate of PD after STN-DBS and to train the machine learning model that can predict the therapy's effect. The functional connectivity analyses suggested that the GBRT model performed best with Pearson's correlations of *r* = 0.65, *p* = 2.58E−07 in medication-off condition. The connections between middle frontal gyrus (MFG) and inferior temporal gyrus (ITG) contribute most in the GBRT model.

## Introduction

Parkinson's disease (PD) is a common neurodegenerative disorder with a wide range of motor and non-motor symptoms, such as cognitive impairment, autonomic dysfunction, disorders of sleep, depression, or hyposmia, which lead to a severe burden for the patients and their caregivers (Poewe et al., 2017). It is considered that PD arises from dysfunction in several neural networks. Thilo van Eimeren et al. confirmed that the medial prefrontal cortex and rostral ventromedial caudate nucleus were functionally disconnected in PD (Thilo van Eimeren et al., 2009). Hammond et al. found that PD patients showed abnormally synchronized oscillatory activity at multiple levels of the basal ganglia (BG)–cortical loop (Hammond et al., 2007).

To cure PD, highly efficacious therapies, such as pharmacological dopamine substitution, have been adapted widely (Poewe et al., 2017). The use of levodopa as dopamine-replacement therapy is highly effective in ameliorating the symptoms of the disease (Fahn et al., 2004) through changing the motor cortex hypoactivation in the supplementary motor area and the primary motor cortex (Buhmann et al., 2003). Deep brain stimulation (DBS) at high frequency was firstly used in 1997 to replace thalamotomy in treating the characteristic tremor of PD and has subsequently been applied to the pallidum and the subthalamic nucleus (STN) (Benabid, 2003). It is reported that neurostimulation of STN was more effective than medical management alone (Deuschl et al., 2006).

DBS therapy is an invasive and costly procedure, and its outcome differs in patients with PD (Cury et al., 2014). While a growing body of research suggests that variability in treatment response links up with individual differences in neurological function (Hartmann et al., 2016), the search for brain network-based biomarkers can yield a reliable indicator for future treatment response in this respect. The identification of brain-based predictors of PD can not only expand existing biological knowledge of neurodegenerative pathophysiology but also inform real-world clinical practice by assignment of patients to make decisions based on individual patterns of neural function or biomarkers.

Nowadays, powerful neuroimaging methods, such as magnetic resonance imaging (MRI), establish accurate and high-precision observation from the view of neuronal activities (Cohen et al., 1993). In particular, the application of functional magnetic resonance imaging (fMRI) in neuroscience has offered a way to assess the status of functional systems, which can reveal relationships between brain activity and treatment response, such as obsessive–compulsive disorder (Figee et al., 2013), depression (Guo et al., 2012), pediatric anxiety disorders (McClure et al., 2007), etc. Neuroimaging studies have also identified impairments in the corticostriatal network pathways and the related neural circuits in patients with PD (Hacker et al., 2012).

Moreover, studies of large-scale network analysis using graph theory-based approaches revealed disruptions in the topological properties of brain networks in PD patients. For example, it was found that PD patients had lower clustering coefficient and local efficiency than control subjects, which can contribute to identifying and tracking PD (Luo et al., 2015). Kim et al. found that PD was related to the temporal properties of brain functional connectivity states as well as the variability of network topological organization using resting state fMRI (rs-fMRI) (Kim et al., 2017). These findings of graph theory-based analysis of fMRI in PD give us insights into the possibility of predicting the outcome after DBS with brain networks.

It is confirmed that specific connectivity profiles encompassing frontothalamic streamlines correlated with clinical response, which can guide surgeons to locate DBS electrode in surgery (Horn et al., 2017). There is also a series of specific patterns of the brain that can enhance the clinical care of DBS, such as frontal white matter architecture in curing major depression (Coenen et al., 2019) and posterior thalamus (Tha) in treating essential tremor (Al-Fatly et al., 2019). With these approaches, surgery can be utilized easily, and the sophisticated relationship between the effectiveness of operation and the intrinsic brain connectome can be discovered.

Machine learning as a data-driven technique can use spatiotemporal information to extract the stable whole-brain patterns that are present in MRI data. Because machine learning is effective in automating the process of building models that relate neural activity to symptoms, it has been attempted to use machine learning for predicting response after DBS (Bermudez et al., 2019; Habets et al., 2019).

In this paper, we aimed at building a model to predict the outcome (percentage change in the Unified Parkinson's Disease Rating Scale (UPDRS)-III score) after DBS through functional brain connectivity. We hypothesized that the outcome of stimulation based on whole-brain networks; thus, functional connectivity profiles would predict the individual outcomes of DBS for PD. The results suggested that the model was capable to predict the DBS outcome, and that the most contributive connections to the prediction were detected.

## Materials and Methods

### Participants and Assessment

This study included 50 patients aged from 50 to 77 (mean age = 60.24 ± 7.84 years) with a final clinic diagnosis of PD. They were recruited from Tsinghua University Yuquan Hospital, Beijing, China, and their disease severities were assessed according to the motor section of the Movement Disorder Society (MDS) UPDRS-III (Antonini et al., 2013). All of them received preoperative MRI and evaluation of dopaminergic responsiveness, and they were considered suitable to DBS surgery according to acute levodopa challenge test (Defer et al., 1999; Rodriguez et al., 2007). The assessing procedure was conducted by a specialist with more than 10 years of experience. All participants were informed about the procedures in this protocol and provided informed consent before the experiment. The research protocol was approved by the Ethics Committee of Tsinghua University Yuquan Hospital.

To be noted, the DBS outcome measure was measured as percentage change in UPDRS-III score comparing postoperative ON DBS to preoperative baseline. The baseline UPDRS-III score was 43.9 ± 12.1, and the UPDRS improvement rate with DBS was 65.2 ± 20.6%.

### Surgical Procedure

DBS surgery was performed under local anesthesia, using the Leksell stereotactic frame (Elekta AB, Stockholm, Sweden). Two STN-DBS electrodes (PINS L301; Beijing, China) were placed in both hemispheres. During the operation, a single unit of microelectrode kept stimulating and recording continuously to evaluate and confirm the site with the best clinical results. After the lead placement was confirmed, the electrodes were connected to a pulse generator (G102R; Pinchi, Beijing, China), which was implanted subcutaneously in the right subclavian region. During surgery, MRI scanning was used for both preoperative targeting and immediate postoperative verification (Foltynie and Hariz, 2010). It was ensured that electrode contacts were well-sited within the STN.

### Image Acquisition

MRI scans were conducted 2–3 days before the operational therapy for all PD patients, and each patient was scanned after withdrawal from levodopa for more than 12 h.

Imaging data were collected on a 3T Philips Achieva MRI scanner (Philips Healthcare, Best, The Netherlands) with a 32-channel head coil. Participants were instructed to keep their eyes open and not to think about anything specific during the rs-fMRI scan. Head motion was controlled by fixing their heads using headphone and sponge during scanning. Resting state blood oxygenation-level-dependent (BOLD) signals were collected using the following parameters: 35 axial slices, repetition time (TR) = 2,000 ms, the number of volumes = 240, echo time (TE) = 30 ms, flip angle (FA) = 90°, slice thickness = 4.0 mm, gap = 0.8 mm, acquisition matrix = 64 × 64, and field of view (FOV) = 224 × 224 mm^2^.

### Image Preprocessing and Brain Network Construction

Whole-brain functional networks were constructed using SPM 12 and GRETNA software (Wang et al., 2015). The following pre-processing steps were taken: (1) the first 10 volumes of each scan were discarded for magnetization equilibration, (2) data were realigned to the first volume to correct for head motions, (3) bottom-up slice-timing correction was applied, (4) functional images were co-registered to subject-space (the same participant's T1-weighted structural image), then spatial normalization was conducted to acquire Montreal Neurological Institute (MNI) template space, and (5) spatial smoothing was performed at 4 mm full-width half maximum (FWHM) Gaussian kernel. According to the Brainnetome Atlas (BNA) (Fan et al., 2016), we segmented the whole brain into 246 regions, including 210 cortical and 36 subcortical regions. Each region served as one node of functional brain networks, and it can also be regard as a region of interest (ROI). The mean time series of each ROI was obtained by averaging the BOLD time series over all voxels within that region. The edges of functional brain networks were computed by Pearson correlation coefficients between ROIs.

The T1-weighted volume MRI data and fMRI data were used for DBS lead localization, and this protocol followed the steps in the manual of Lead-DBS (Horn and Kühn, 2015). Images were normalized into ICBM 2009b NLIN asymmetric space using the DISTAL Minimal atlas (Ewert et al., 2017), and DBS electrode contacts were localized within MNI space using Lead-DBS software (www.lead-dbs.org) (Horn and Kühn, 2015).

### Connectome-Based Predictive Modeling

According to the BNA, we acquired 30,135 connectivities between ROIs, and the dimension space of connectivity matrix is so large that it can lead to a serious overfitting problem. Therefore, feature preparation was conducted on connectivity between ROIs. To be more specific, we narrowed down the feature space of sparse matrixes through random forest algorithm, which is a multivariate supervised approach that can retain essential pre-surgical features.

**Figure 1:**
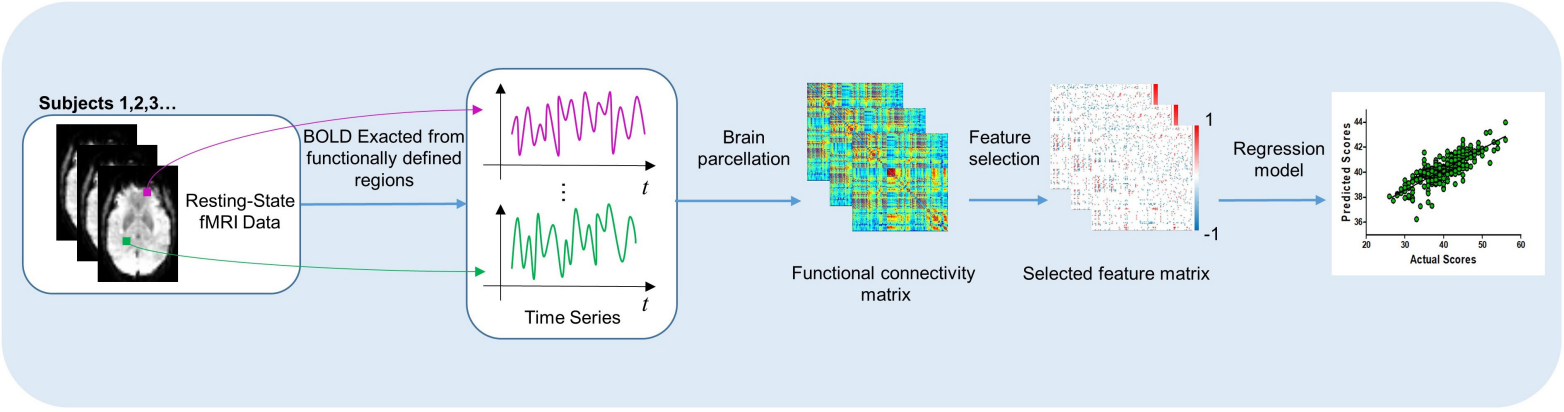
The process of our work, which includes learning the significant features in functional brain network and predicting the outcome after DBS.

As shown in Figure 1, our process of learning and predicting mainly includes four parts: (1) all participants were scanned by an MRI scanner to acquire BOLD time series in rs-fMRI, (2) the functional connectivity network was constructed through computing the Pearson correlation coefficients between ROIs, (3) feature selection was applied, and (4) use machine learning method to train the predictive model.

Six predictive models were implemented in this study, including linear regression models with Ordinary Least Squares (OLS) (Goldberger, 1964), ridge regression (Tibshirani, 1996b), or least absolute shrinkage and selection operator (lasso) (Tibshirani, 1996a) and non-linear regression models with Support Vector Regression (SVR) (Drucker et al., 1997), Gradient Boost Regression Tree (GBRT) (Friedman, 2001), or reformed random forest named Extremely Randomized Trees (ERT) (Geurts et al., 2006). We used nested cross-validation, which included outer Leave-One-Out-Cross-Validation (LOOCV) and inner 5-fold cross-validation (5F-CV), to quantify the prediction accuracy. The inner 5F-CV was used to determine the optimal parameters (e.g., α, λ) for six machine learning algorithms, and outer LOOCV was applied to evaluate the generalizability of the model.

In the inner 5F-CV, we used grid search method to find the best estimator for six models and evaluated each estimator by measuring the prediction error of the model. Then, we acquired six models with suitable estimator to predict the outcome of DBS surgery and choose the most predictive model to conduct connection analysis accordingly.

Because the dataset size is limited compared with tens of thousands of features in PD patients' brain, a Leave-One-Out-Cross-Validation (LOOCV) was used in the outer loop to maximize the prediction model to learn existing data (Kohavi, 1995). In the LOOCV, one sample was used as validation data, and the other samples were used as training data. In the dataset with n subjects, the data of n−1 subjects were used as input to train the model, and this process was repeated n times with different left-one-out subjects, generating the estimated percentage changes of UPDRS-III score, identified functional connectivity and their corresponding weights in the training model. This allows us to investigate the biological characteristic of these connections between ROIs by analyzing important connections and nodes chosen by machine learning model.

## Results

### DBS Lead Placement

**Figure 2:**
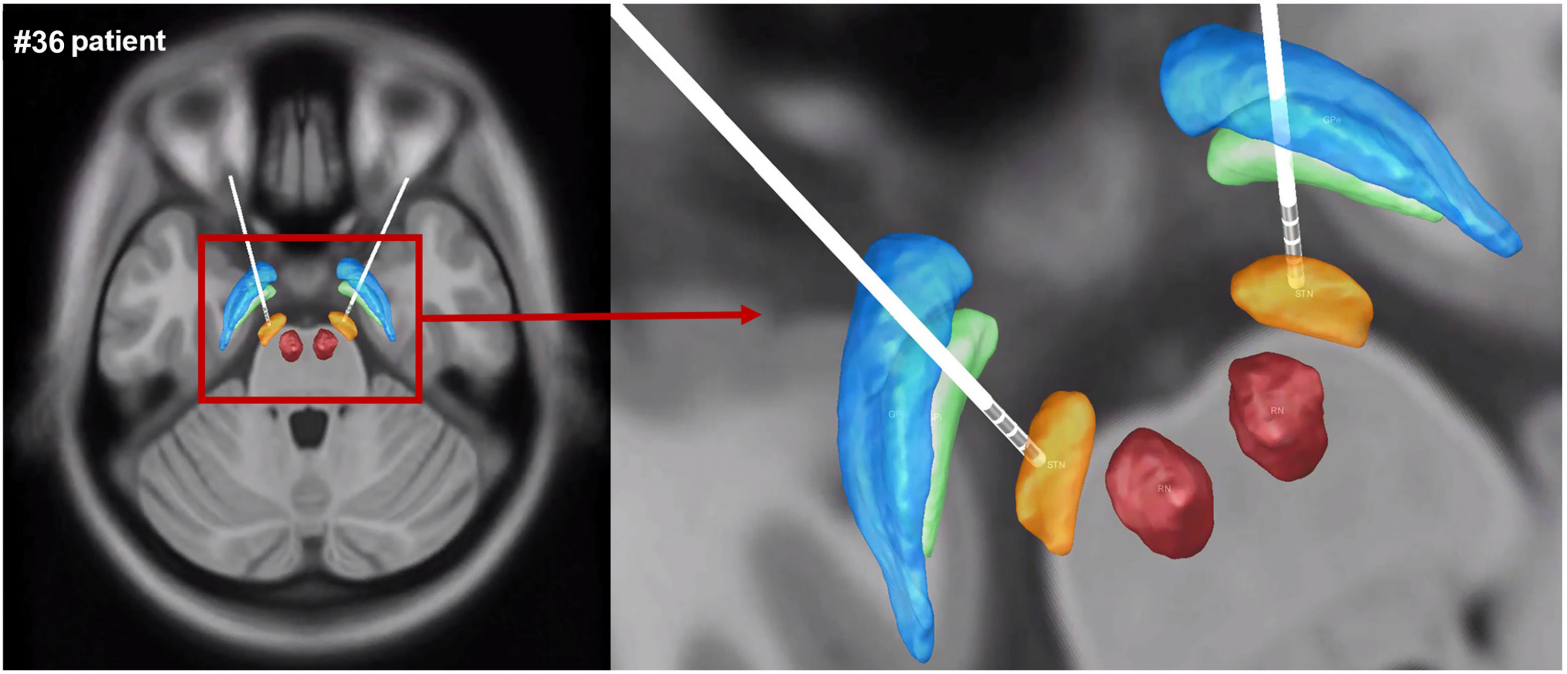
The reconstruction image of #36 patient's DBS lead localization. Gpe, globus pallidus externus; GPi, globus pallidus internus; STN, subthalamic nucleus; RN, red nucleus (blue: GPe, green: GPi, orange: STN, red: RN).

The electrode contacts were well-sited within the STN, and DBS lead localization was reconstructed using Lead-DBS. The reconstruction image of #36 patient was shown as an example in Figure 2.

### Feature Selection and Connection Analysis

By choosing BNA-based functional connectivity matrix, we acquired 30,135 pairs of connections by removing the repeated connections from the 246 × 246 combinations in whole-brain connections for each subject. As mentioned in the Materials and Methods section, we used random forest to exclude redundant connections. Finally, the data showed that there were 242 connections for predicting the outcome of DBS without levodopa.

### Predicting the Individual Outcome of DBS With PD

**Figure 3:**
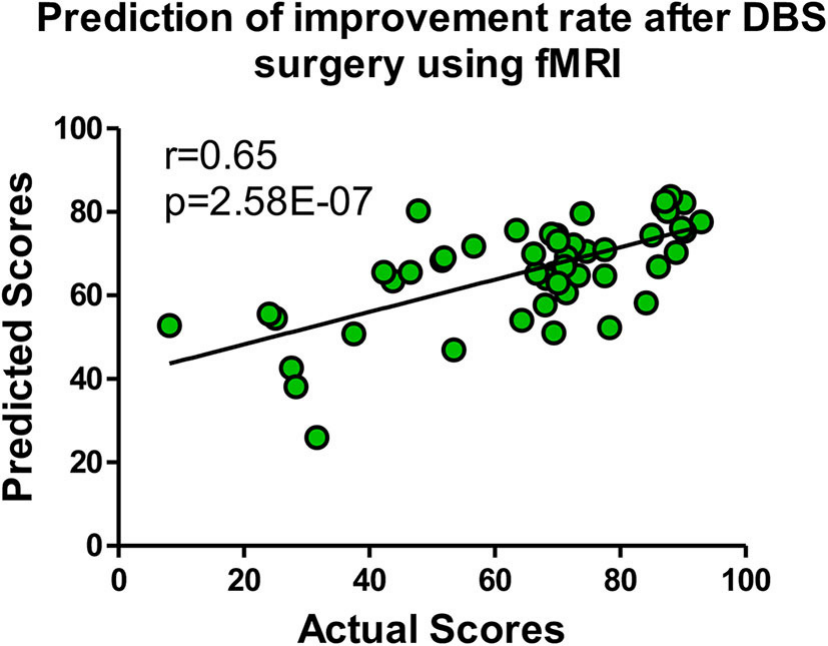
The GBRT model with the most predictive performances of Pearson correlations *r* = 0.65, *p* = 2.58E−07 in medication-off condition.

Six models (OLS/ridge regression/lasso/SVR/GBRT/ERT) were implemented for the prediction of the DBS outcome. To test the reliably of our connectome-based predictive model, we employed an outer LOOCV analysis to predict the improvement rate in UPDRS-III score after DBS. Four indicators [i.e., Pearson's *r*, Pearson's *p*, mean absolute error (MAE), and mean square error (MSE)] were utilized to measure the performance of each predicting model, shown in Table 1. Pearson's r is an indicator to measure the correlations between two objects, whereas Pearson's *p*-value corresponds to a test for whether the correlation was significantly different from zero (*p* < 0.05 was considered statistically significant). We also used MAE (Willmott and Matsuura, 2005) and MSE (Imbens et al., 2005) to describe the average model-performance error. The correlations between predicted percentage change in UPDRS-III score and actual percentage change in UPDRS-III score were significant in our model based on functional connectivity. The best fitting model came from the GBRT model with Pearson correlations of *r* = 0.65, *p* = 2.58E−07 in medication-off condition, shown in Figure 3. In addition, the Bonferroni correction (Abdi, 2007) was used in performing multiple tests, and the Pearson's p was less than the stricter threshold of 0.001.

**Table 1:** Performance of six models in predicting the improvement rate in UPDRS-III score by using nested cross-validation.

**Model**	**MAE**	**MSE**	***r***	***p*-value**
OLS	21.14	862.94	0.05	0.75
Ridge regression	17.57	573.95	0.14	0.35
Lasso regression	14.29	411.83	0.33	0.02
GBRT	12.40	240.74	0.65	2.58E−07
SVR	16.06	398.57	0.28	0.05
ERT	13.12	282.13	0.59	6.67E−06

### Connections Contributing to Prediction

**Figure 4:**
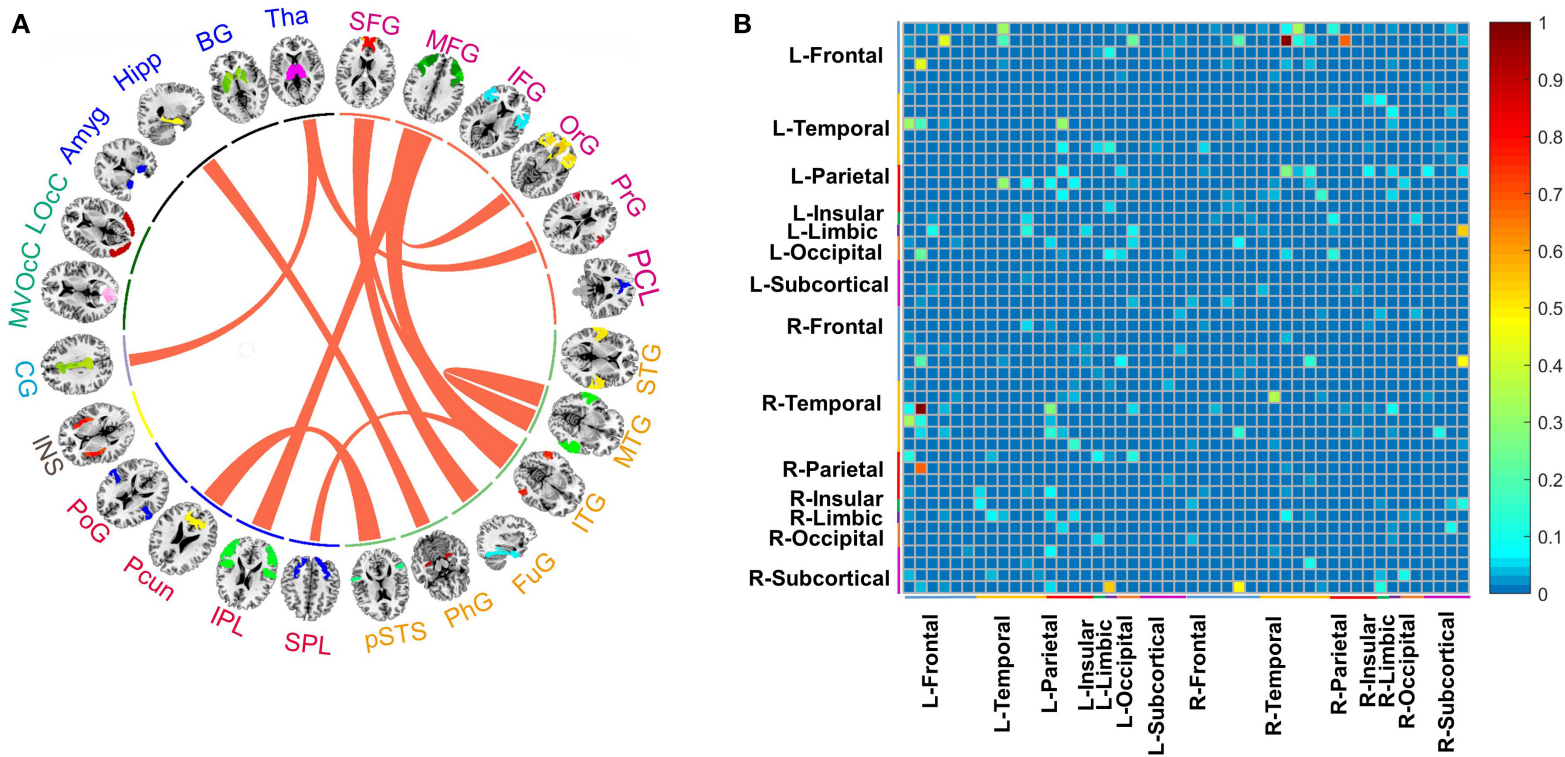
**(A)** The top 11 predictive connections of 24 macroscales brain designed by BNA in medication-off condition. **(B)** The distribution of predictive connections selected by the GBRT model without levodopa, which is divided into the left and right brain hemispheres. The range of color bar in **(B)** is from 0 to 1, and it represents the importance of connections between regions in prediction.

Based on stable prediction, further brain analysis could be conducted by the GBRT model. For better interpretation, we grouped the 246 ROIs into 24 gyri as defined by BNA and calculated the top 11 predictive connections between 24 gyri, shown in Figure 4A. The gyri of each brain hemisphere were further divided into five lobes, and the predictive connections selected by the GBRT model from the perspective of the lobes were shown in Figure 4B.

In predicting the outcome of DBS without levodopa, middle frontal gyrus (MFG), inferior temporal gyrus (ITG), superior frontal gyrus (SFG), and Tha show more connections than other regions. The top 11 predictive connections were shown in Table 2. The connections of cross-brain regions, such as MFG and ITG, precuneus (Pcun), and posterior superior temporal sulcus (pSTS), exerted an enormous function on medication-off condition.

**Table 2:** The top 11 connections in the prediction of improvement rate in UPDRS-III score after the deep brain stimulation operation in medication-off condition.

**ID**	**Node name**	**ID**	**Node name**
1	Superior frontal gyrus (SFG)	9	Inferior temporal gyrus (ITG)
1	Superior frontal gyrus (SFG)	10	Fusiform gyrus (FuG)
2	Middle frontal gyrus (MFG)	4	Orbital gyrus (OrG)
2	Middle frontal gyrus (MFG)	9	Inferior temporal gyrus (ITG)
2	Middle frontal gyrus (MFG)	14	Inferior parietal lobule (IPL)
5	Precentral gyrus (PrG)	24	Thalamus (Tha)
8	Middle temporal gyrus (MTG)	8	Middle temporal gyrus (MTG)
9	Inferior temporal gyrus (ITG)	13	Superior parietal lobule (SPL)
11	Parahippocampal gyrus (PhG)	22	Hippocampus (Hipp)
12	Posterior superior temporal sulcus (pSTS)	15	Precuneus (Pcun)
18	Cingulate gyrus (CG)	24	Thalamus (Tha)

*1–6: fontal, 7–12: temporal, 13–16: parietal, 17: insular lobe, 18: limbic lobe, 19–20: occipital lobe, 21–24: subcortical nuclei*.

## Discussion

The actual outcome of PD patients was based on their motor and non-motor symptoms. To assess the condition of PD patients, there were the Hoehn and Yahr (H&Y) scale (Ramaker et al., 2002) for quantifying disease stage, MDS-UPDRS (Goetz et al., 2008) for assessing the patient's condition clinically, Beck Depression Inventory (Beck et al., 1988) for measuring the patient's degree of depression, and Mini-Mental State Examination (Folstein et al., 1983) for intellectual impairment. The objective of this study was to explore the relationship between brain connectivity and DBS outcome regarding the motor symptoms among PD patients. UPDRS-III provides a useful severity measure on the motor symptoms of PD (Tison et al., 2002), and it was reliable (Metman et al., 2004). Therefore, the DBS outcome in this study was measured as the percentage change in UPDRS-III score.

Based on the results of 50 PD patients in this study, we were able to characterize networks that can predict the recovery after the DBS therapy. These network features played significant roles in training the machine learning model. In PD patients treated by DBS without levodopa, the connections of the MFG to ITG, Pcun to pSTS, and internal connection in middle temporal gyrus (MTG) were found to provide top contribution in the GBRT model to the prediction of operation therapy. These findings provide new evidence that the functional connectivity has an effect on predicting the DBS operation outcome in PD patients before the operation. This progress may potentially help reduce the loss of money and the trauma of body in patients with unsatisfactory DBS response (Ellis et al., 2008).

As a white matter lesion associated with motor and cognitive symptoms (Gattellaro et al., 2009), PD is related to topological properties (Olde Dubbelink et al., 2013), through which the effectiveness of DBS can be assessed (i.e., global efficiency, clustering coefficient, and small-worldness) (van Hartevelt et al., 2014). Moreover, the predictive value of connectivity-informed brain stimulation for DBS can be seen in obsessive–compulsive disorder (Baldermann et al., 2019), resistant depression (Johansen-Berg et al., 2007), and tremor disorder (Middlebrooks et al., 2018). These results indicated that it might be feasible to predict the outcome of PD patients treated by DBS.

There are already previous studies related to PD that present results similar to our work. Brain activity in the right ITG and MFG was also found related to gait in PD (Wang et al., 2016). Comparing with healthy controls, PD patients showed increased functional connectivity in ITG (Yang et al., 2016). Furthermore, Grafton et al. found that effective DBS can smoothen the overactivity in bilateral rostral ITG of PD patients toward a more normal pattern (Grafton et al., 2006), which suggested that the pattern of ITG may be a biomarker indicating the outcome of DBS. It was reported that gray matter atrophy or cortical thinning in MFG is related to PD (Brenneis et al., 2003; Biundo et al., 2015), and that it can also be a predictor of conversion to dementia in PD patients (Song et al., 2011). These findings were consistent with our results that ITG and MFG showed more connectivity with other gyri and the connections between ITG and MFG have significant contribution in the model predicting the outcome of DBS in medication-off condition.

It has been confirmed that PD patients exhibited decreased short-range functional connectivity densities in SFG (Zhang et al., 2015). SFG is one of the most important gyri for executive control (Kendi et al., 2008), and cortical atrophy in SFG can affect the motor cortex (Possin et al., 2013). Similar to SFG, Pcun is also associated with network modulation in the treatment of PD patients. In PD patients, the functional connectivity between Pcun and motor system is decreased (Thibes et al., 2017), and the metabolic in Pcun increased after STN-DBS according to the study based on PET (Asanuma et al., 2006). The association between DBS outcome and SFG and Pcun is congruent with the results of prior studies.

The current study indicated that the frontal lobe and temporal lobe play an important role in predicting DBS's effect. Among the top 11 predictive connections, there are one or both ends of 17 connections distributed in the frontal lobe and temporal lobe. Kostić et al. have also found that a specific pattern of brain network damage involving the frontal and parietal cortices occurs in patients with freezing of gait (Kostić et al., 2012). It was also reported that a lack of adequate frontal activation was found to be related to PD patients (Jahanshahi et al., 2010), and that the modulation by STN-DBS was found to be correlated to the suppression of alpha and beta oscillations in the temporal area based on a MEG study (Cao et al., 2017). By comparing six machine learning models, the GBRT regression model was able to estimate the improvement rate of UPDRS-III score after DBS most accurately. The GBRT regression model (Friedman, 2001) is an ensemble of weak prediction models (decision trees) based on gradient boosting. GBRT sequentially adds small trees (low depth) with high bias, so that it can better fit target. It has been widely used in many fields of regression problems because of its high prediction accuracy. To conclude, GBRT regression offers many advantages over the traditional multiple-regression models, with the ability of processing non-linear data. Besides the GBRT model, the ERT model also showed excellent prediction. In the two collections of top 11 connections selected by the GBRT and ERT regression models, respectively, 10 connections were the same (Supplementary Table 1), which also verified the accuracy of the GBRT model in prediction.

It has several limitations when interpreting the findings in our study. First, there are some factors that influence variables during the operation, such as surgical instruments, doctors' operations, etc. These factors have not been fully considered. Second, due to the difficulty in obtaining the data clinically, the amount of sample is still small from the perspective of machine learning, which may cause errors. In further research with larger dataset of more PD patients carried out DBS surgery, more predictive patterns can be found, and there can be more comprehensive evaluation before surgery.

## Conclusion

In this study, we investigated the relationship between functional connectivity and outcome of DBS therapy in 50 PD patients. Using machine learning models, we demonstrated that the functional network can predict the outcome of operation therapy. The GBRT model is the most effective machine learning model with Pearson correlations *r* = 0.65, *p* = 2.58E−07 in medication-off condition, and the most contributable connections for models were identified.

## Data Availability Statement

The datasets generated for this study are available on request to the corresponding author.

## Ethics Statement

The studies involving human participants were reviewed and approved by the Ethics Committee of Tsinghua University Yuquan Hospital. The patients/participants provided their written informed consent to participate in this study.

## Author Contributions

XL, YM, and RS conceived the research project. XL supervised the research project. LH and YM processed the raw Parkinson's disease dataset. XM, XL, and RS conducted the computational analyses. RS, XM, and XL wrote this manuscript. All the authors discussed the experimental results and commented on the manuscript.

## Conflict of Interest

The authors declare that the research was conducted in the absence of any commercial or financial relationships that could be construed as a potential conflict of interest.
