# Supplementary material for: Connectome-Based Model Predicts Deep Brain Stimulation Outcome in Parkinson's Disease
Source: Front Comput Neurosci. 2020 Oct 28;14:571527. doi: 10.3389/fncom.2020.571527 (PMC7656054; doi:10.3389/fncom.2020.571527)
Supplement: Supplementary file 1 [file Presentation_1.PDF]

Supplementary Material

1 Supplementary Data

1.1 Top predictive connections detected by ERT

Extra Trees regression (ERT, (Geurts, Ernst, & Wehenkel, 2006)) is tree-based ensemble method for supervised classification and regression problems. Based on prediction of ERT model (Pearson's correlations of r = 0.59, p = 6.67E-06), we also conducted further brain analysis. We grouped the 246 ROIs into 24 gyri defined by BNA, and calculated the top 11 predictive connections between 24 gyri, shown in Supplementary Figure 1(A). The gyri of each brain hemisphere was further divided into 5 lobes, and the predictive connections selected by ERT model from the perspective of lobes were showed in Supplementary Figure 2(B).

1.2 Data access statement

Our data is publicly available for all researchers. But in order to prevent data from being misused, all researchers who try to obtain experimental data need to apply to the corresponding author of this paper through e-mail. The application should include the full name, affiliation, a short description of purpose and a statement that guarantees that the data will only be used for research, study and teaching purposes, and will not attempt to infer the true identity of the subject based on the data.

2 Supplementary Figures and Tables

2.1 Supplementary Figures

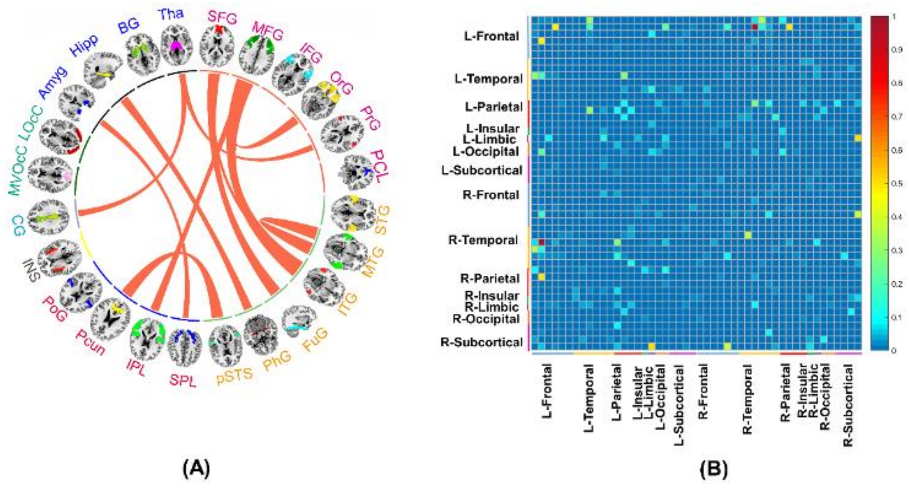

Supplementary Fig. s3. (A)The top 11 predictive connections of 24 macroscale brain designed by BNA in medication-off condition. (B) the distribution of predictive connections selected by ERT

model without levodopa, which is divided into left and right brain hemisphere. The range of colorbar in (B) is from 0 to 1, and it represents the importance of connections between regions in prediction.

2.2 Supplementary Tables

Supplementary Table s4. The same 10 connections selected by ERT and GBRT model in the prediction of improvement rate in UPDRS-III score after DBS in medication-off condition.

ID	Node name	ID	Node name
1	Superior Frontal Gyrus (SFG)	10	Fusiform Gyrus (FuG)
2	Middle Frontal Gyrus (MFG)	4	Orbital Gyrus (OrG)
2	Middle Frontal Gyrus (MFG)	9	Inferior Temporal Gyrus (ITG)
2	Middle Frontal Gyrus (MFG)	14	Inferior Parietal Lobule (IPL)
5	Precentral Gyrus (PrG)	24	Thalamus (Tha)
8	Middle Temporal Gyrus (MTG)	8	Middle Temporal Gyrus (MTG)
11	Parahippocampal Gyrus (PhG)	22	Hippocampus (Hipp)
12	posterior Superior Temporal Sulcus(pSTS)	15	Precuneus (Pcun)
13	Superior Parietal Lobule (SPL)	21	Amyg (Amygdala)
18	Cingulate Gyrus (CG)	24	Thalamus (Tha)

1-6: Fontal 7-12:Temporal 13-16:Parietal 17: Insular Lobe 18: Limbic Lobe 19-20: Occipital Lobe 21-24: Subcortical Nuclei.

3 References

Geurts, P., Ernst, D., & Wehenkel, L. J. M. L. (2006). Extremely randomized trees. *63*(1), 3-42.
